# Dentin bond strength and degree of conversion evaluation of experimental self-etch adhesive systems

**DOI:** 10.4317/jced.51842

**Published:** 2015-04-01

**Authors:** Fatemeh-Maleknejad Yazdi, Horieh Moosavi, Mohammad Atai, Mahsa Zeynali

**Affiliations:** 1 Professor, Dental Materials Research Center, Department of Operative Dentistry, Mashhad Dental School, Mashhad University of Medical Sciences, Mashhad, Iran; 2 Associate Professor, Dental Materials Research Center, Department of Operative Dentistry, Mashhad Dental School, Mashhad University of Medical Sciences, Mashhad, Iran; 3 Department of Polymer Sciences, Iran Polymer and Petrochemical Institute, P.O. Box 14965/159, Tehran, Iran; 4 Assistant Professor, Dental Materials Research Center, Department of Operative Dentistry, Mashhad Dental School, Mashhad University of Medical Sciences, Mashhad, Iran

## Abstract

**Background:**

The aim of this study was to investigate the effect of different concentrations of 10-methacryloyloxydecyl dihydrogen phosphate (10-MDP) monomer in one-step self-etch experimental adhesives on dentinal microshear bond strength (µSBS), their degree of conversion and bonded micro structure.

**Material and Methods:**

Composite resin cylinders (Clearfil AP-X) were bonded on human sound molar dentinal surfaces by using five experimental one-step self-etching adhesives (1-SEAs) containing 0% (E0), 5% (E5), 10% (E10), 15% (E15), 20% (E20) (by weight) 10-MDP monomer and Clearfil S3 Bond (CS3) as a control. After 24 hours, microshear bond strength was tested. The degree of conversion was also measured using Fourier transform infrared spectroscopy. Interfacial ultrastructure was observed under a scanning electron microscope in all the groups.

**Results:**

A higher microshear bond strength was observed with adhesives containing 10% and 15% 10-MDP in comparison to study groups (P<.05). Clearfil S3 Bond and 10% MDP had a significantly greater degree of conversion than other groups (P<.05).

**Conclusions:**

The amount of functional monomer in 1-SEAs influences both the bonding performance and degree of conversion; 10% 10-MDP showed the best combination of bond strength and degree of conversion.

** Key words:**Self-etch adhesives, 10-MDP, bond strength, degree of conversion.

## Introduction

Self-etch adhesives have been promising to overcome the sensitivities associated with etch-and-rinse adhesive systems. They are claimed to be less technique-sensitive and time-consuming and produce an efficient hybrid layer by simultaneous demineralization of and penetration into dentin. In addition, less post operative sensitivity has been reported with these systems (1,2).

Mild self-etch adhesives demineralize dentin only partially, leaving some hydroxyapatite (HAp) crystals around collagen fibers available for chemical bonding to special functional monomers (3,4). However, the chemical interaction of these adhesives with the enamel and dentin depends on the concentration of functional monomers in the primer (5). The adhesion-decalcification concept states that the functional monomers first ionically interact with calcium in HAp and then either decalcify or bond to tooth substrates depending on the stability of the calcium-monomer complex (4,6,7). Methacryloyloxydecyl dihydrogen phosphate (MDP) is one of the best functional monomers present, which is capable of establishing an ionic bond to HAp in a short clinical time (8). MDP‒calcium bond is hardly soluble in water, creating a stable bond on the basis of Yoshida’s adhesion-decalcification concept (4).

1-SEAs are complicated mixtures of different components. There are a number of concerns expressed about this generation of adhesives; phase separation as a consequence of the immiscibility of water with hydrophobic monomers (9) that affects their polymerization, entrapment of water within the interface (10,11), and water diffusion through the smear layer of these adhesives decrease the bond strength (12,13). Incomplete polymerization of adhesives compromises the quality of smear layer and jeopardizes the bond strength and creates a source of uncured monomer release (2,14,15).

Several solutions are proposed for the aforementioned problems such as reducing the water and acidic functional monomer concentration of the adhesives (12). It was definitude that water-free adhesives can reach the bond strength of water-containing all-in-one adhesives if special considerations for keeping the moisture of dentin are taken (16). Various techniques are employed to determine the degree of conversion (DC%) of adhesive resins. Fourier transform infrared (FTIR) spectroscopy is a powerful and reliable method, based on molecular vibrations that can be used to assess the degree of conversion (17).

According to the best of the authors’ knowledge, the literature review does not bring up many studies on the effect of various concentrations of MDP monomer in adhesive systems and its effect on bond strength and degree of conversion. Therefore, this study was undertaken to compare dentin bonding efficacy and degree of conversion of five experimental all-in-one water-free self-etch adhesive systems containing different concentrations of MDP monomer. The null hypothesis tested was that experimental self-etch adhesives are not different in bond strength, degree of conversion and the micromorphology of the bonding interface.

## Material and Methods

**Figure 1:**
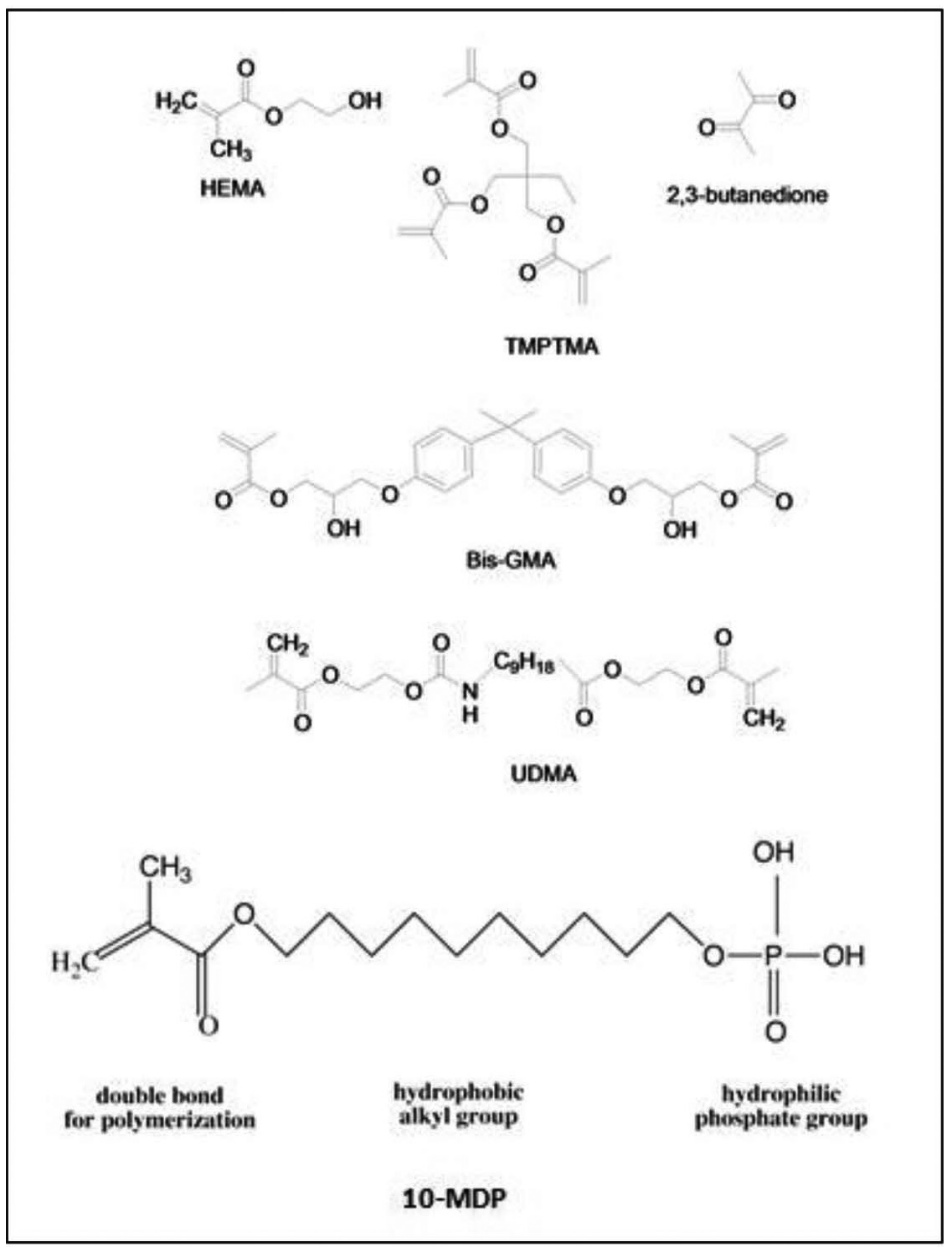
Chemical structure of monomers used in the experimental adhesive resin systems.

The components and application modes of Clearfil S3 Bond (Kuraray Medical Inc. Okayama, Japan) and five experimental groups of 1-SEAs are illustrated in  Table 1 and figure 1. Clearfil S3 Bond was used as the control to compare the performance of the experimental adhesives with it.

**Table 1:**
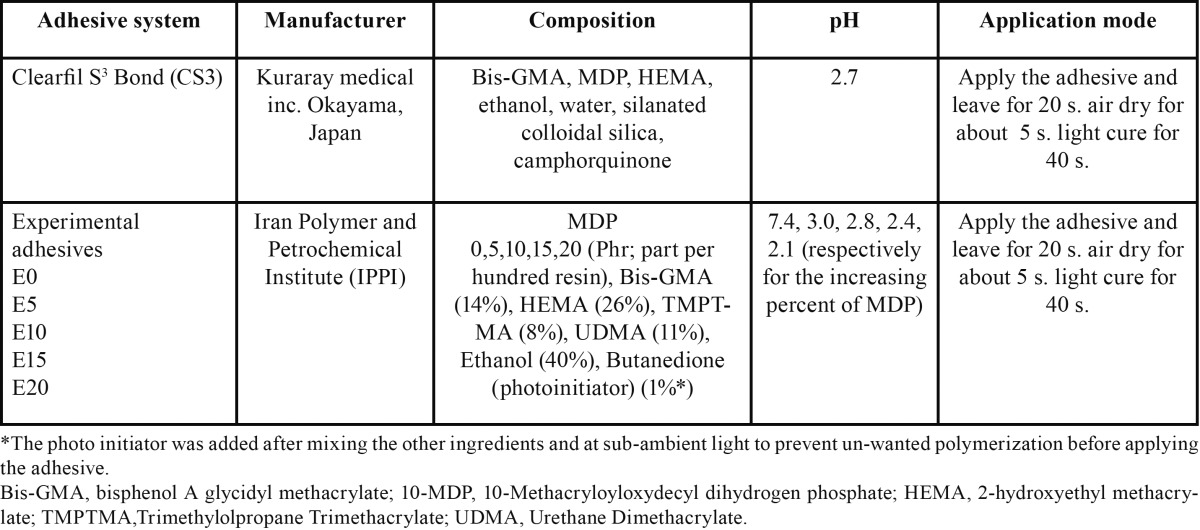
Composition, pH and instructions of use the commercial and experimental adhesive groups.

-Microshear bond strength test

**Figure 2:**
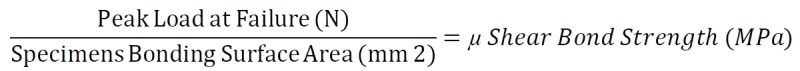
Formula.

Forty‒eight sound human molars were collected under a protocol approved by the Ethics Committee of Mashhad University of Medical Sciences (900511/2011). The teeth were immersed in 2.5% formalin for one week. The teeth were then cleaned of any soft tissues and transferred into distilled water for up to two months. Two 2-mm-thick slices from each tooth were obtained by the crowns being sectioned perpendicular to the tooth long axis using a water-cooled slow-speed diamond saw (CNC, Nemo, Mash-had, Ir). In each slice only the surface close to the occlusal surface with a dentin substrate was used. The flat surface of each slice was then polished with 600 and 1000 grit silicon carbide papers (Soft Flex, Germany) under running water to create a homogeneous smear layer. Ninety-six prepared dentin slices were randomly divided into six test groups. The surfaces were examined under X10 magnifications using a stereomicroscope (Dino-Lite Pro, AnMo Electronics Crop, Taiwan) to exclude any specimens with defects and then treated with the six adhesive systems according to the manufacturer’s instructions for Clearfil S3 Bond. In the control group (Clearfil S3 Bond) dentin surfaces were air-dried before adhesive application, but for the experimental water-free adhesives the dentin surfaces were just blot-dried and the adhesives containing 0, 5, 10, 15, and 20 wt.% of MDP were applied by actively rubbing using a micro-brush for 20 seconds; then the solvents were gently evaporated using an air stream from a 5-mm distance to the tooth surface for about 5 seconds until a homogeneous shiny layer was observed on the surface. During production of the six experimental adhesives, their pH was measured by a pH meter (InoLab, WTW, Germany). Silicon tubes with an internal diameter of 0.7 mm and a height of ~-1.5 mm were placed on dentin near the DEJ of occlusal surface and then the adhesives were light-cured with an LED Bluephase (IvoclarVivadent, Lichtenstein) with a light intensity of 600 mW/cm2 for 40 seconds. A hybrid resin composite, Clearfil AP-X (A3, Kuraray, Japan) was placed into the tubes in two layers and pressed gently and each layer was light-cured for 40 seconds. The specimens were then stored in distilled water at room temperature for 24 hours. Then the tubes were removed using a surgicaly blade. Each tooth slice was attached to the testing apparatus with a cyanoacrylate adhesive and tested in a universal testing machine. A thin steel wire with a diameter of 0.2 mm was looped around each resin cylinder making contact with the lower half-circle of the cylinder and touching the tooth surface. The shear force was applied by pulling the wire loop up at a cross-head speed of .5 mm/min using a universal testing machine (STM, Santam, Iran) until failure occurred. The maximum load required to detach the cylinders from the tooth surface was divided by the bonded surface area and recorded as the microshear bond strength using the formula below: (Fig. 2).

After debonding, the samples were examined under a stereomicroscope (Dino-Lite Pro, Anmo Electronics Corp, Taiwan) at ×50 magnification and the failure modes were defined as adhesive (ADR), cohesive in composite resin or dentin (CD) and mixed (M).

-SEM evaluation

Three specimens in each group at the cross–sectional composite–dentin interface were analyzed using SEM (SEM, LEO1450 VP, Germany). The samples were mounted on the aluminum stub and then coated with gold for 120 seconds using a sputter coater (Polaron Sputter Coater, UK).

Degree of conversion test

**Figure 3:**
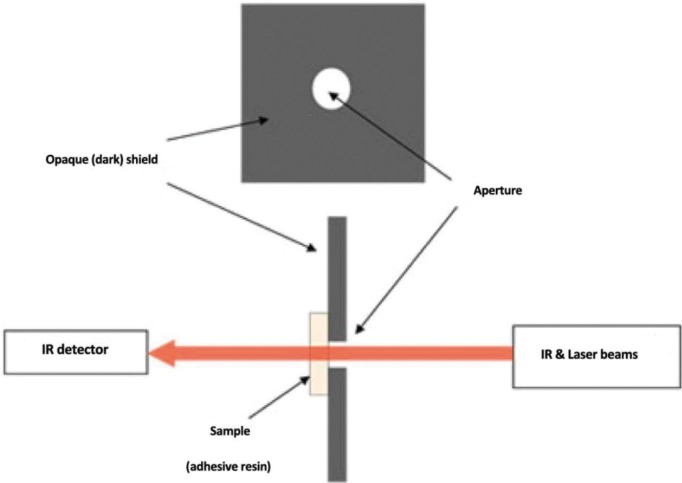
Set-up for measuring degree of conversion of adhesive resins by Fourier transforms infrared (IR) spectroscopy.

**Figure 4:**
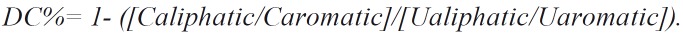
Formula.

For FTIR spectroscopic analysis, equal droplet amount of each adhesive resin was placed on a transparent poly-ethylene film.With a gentle steam of air, the solvents were evaporated for 30 seconds and then covered with a second film and pressed softly to form a thin layer of the adhesive. The “sandwich” was placed into the sample holder of FTIR spectrometer (Equinox 55, Bruker, Germany) (Fig. 3), and the absorbance peaks of the unpolymerized adhesives were recorded by transmission mode at a resolution of 4 cm-1, with scans in the range of 400-4000 cm-1. The adhesives were then light–cured with an LED Bluephase (IvoclarVivadent, Lichtenstein) light–curing unit with a light intensity of 600 mW/cm2 for 40 seconds and the absorptions were recorded for the cured adhesive specimens. The DC% was calculated from the ratio of absorbance intensities for aliphatic C=C (peak at 1638 cm-1) and the internal reference of aromatic carbon-carbon double bonds (peak at 1608 cm-1) were recorded before and after curing the specimens, according to the following equation: (Fig. 4)

in which ‘C’ refers to cured and ‘U’ refers to uncured adhesive.

-Statistical analysis 

Kolmogorov-Smirnov test indicated using the parametric test for data analyzed (*P*>.05). Data were analyzed using one-way-ANOVA and the Tukey HSD test at the significance level of .05.

## Results

-Microshear bond strength (µSBS)

The maximum and minimum values of mean µSBS were observed in E10 and CS3 groups respectively ( Table 2). ANOVA sho-wed a significant difference in µSBS mean of the tested adhesive resins (*p*<.05). Comparisons between groups showed this signi-ficant difference among the E10 and E15 groups with the others ( Table 2).

**Table 2:**
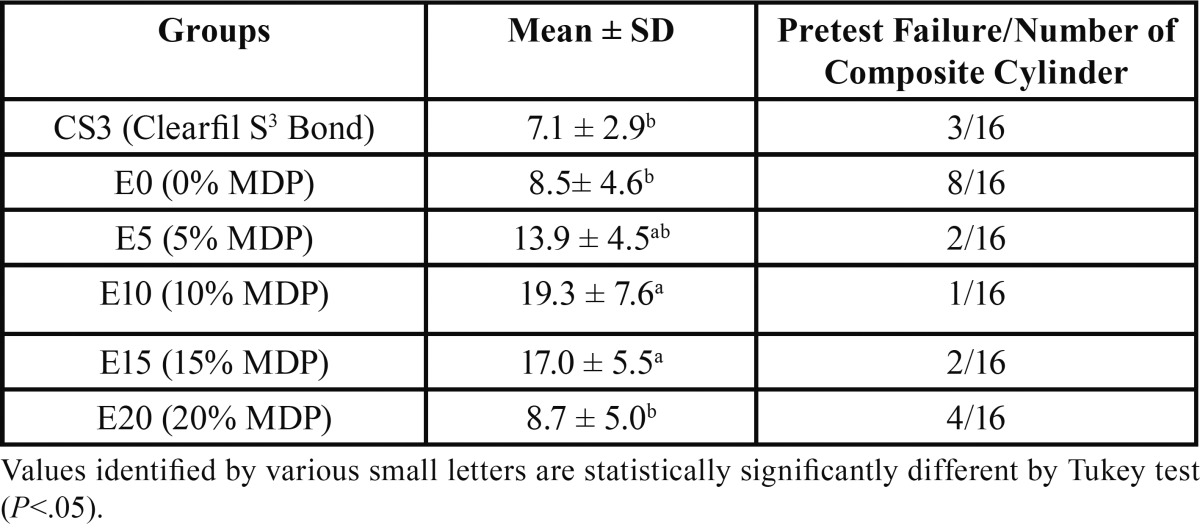
Microshear bond strength values (in MPa± SD) of tested self-etch adhesive systems.

In terms of failure mode, the adhesive failure mode was the most prevalent failure mode in all the study groups ( Table 3).

**Table 3:**
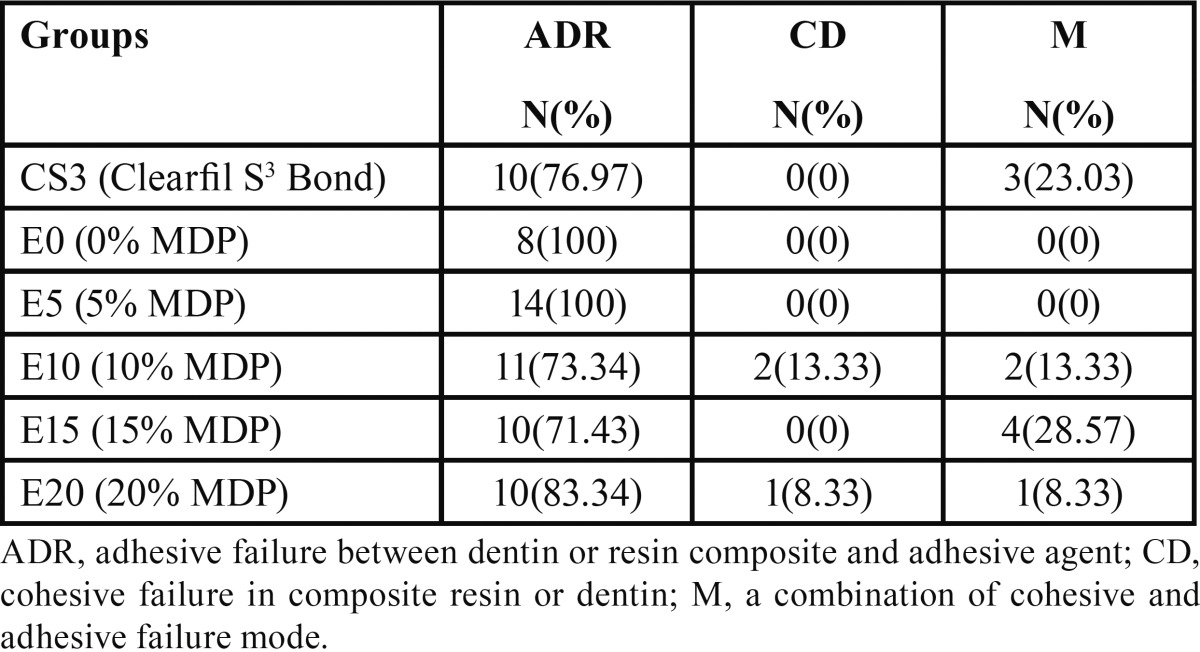
The distribution of failure mode in the study groups.

-SEM 

**Figure 5:**
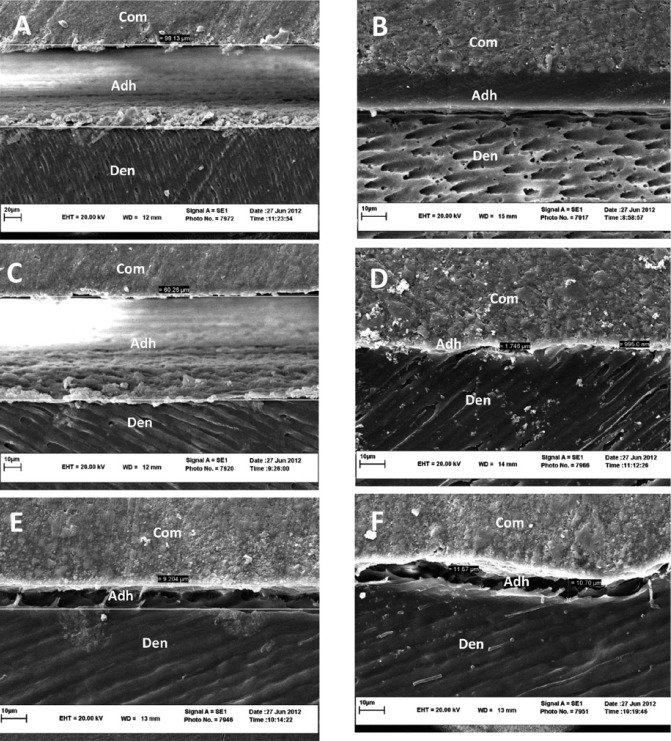
SEM micrographs of bonded interface of adhesives to dentin (×3000, bar=10µm). A) 0% MDP, unmodified smear layer remained on dentin surface and large gaps are noticed. B) Clearfil S3 Bond shows a good consistency between composite, adhesive and dentin surface. C) 5% MDP, residual smears and lack of adhesion is clearly observed. D) 10% MDP, proper interfacial consistency with only submicron sized gaps are observed. E, F) 15% and 20% MDP, similar SEM images with almost the same size gaps.

An interfacial gap was observed in SEM images of all the adhesives except for CS3 and the adhesive containing 10% MDP mo-nomer (Fig. 5).

Degree of conversion (DC %)

The highest and the lowest DC% were observed for the CS3 and E0 groups respectively ( Table 4). It was revealed a significant difference existing in DC% of the examined adhesive resins (*p*<.05). The results of group pairwise comparison are displayed in  Table 4.

**Table 4:**
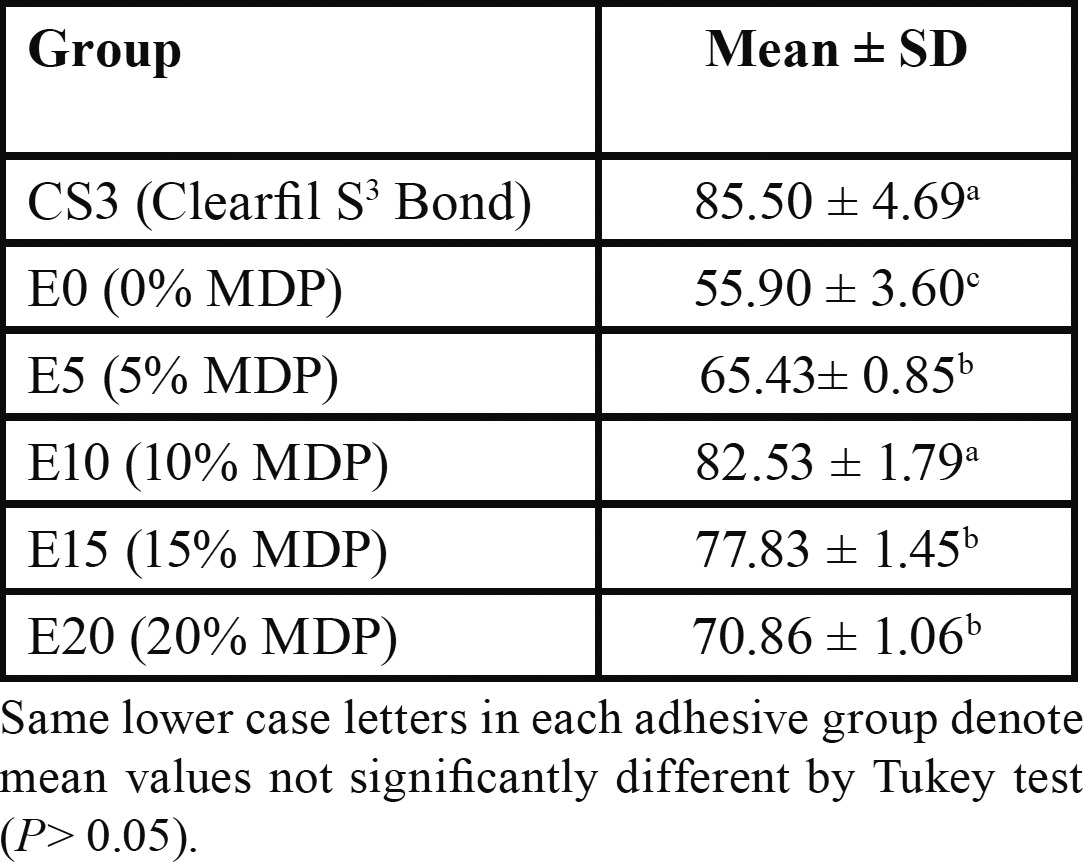
Degree of conversion for tested self-etch adhesive systems (mean± SD; n = 5).

## Discussion

A durable and high bond strength to dentin is the goal of all the restorative materials and procedures. The bond strength is measured with different methods among which microshear bond strength is a simple and reliable method (18). However, one of the reasons that µSBS test is not commonly used is covering the whole surface with the adhesive resin. Therefore, the bonding area is not defined clearly (probably larger than expected) and the stress report may be unreliable as a result (19). However, when this test is combined with DC or other properties of the adhesive itself, it might pose fewer problems, similar to that in this study.

Clearfil S3 Bond which is an MDP-containing all-in-one adhesive, had a formulation most similar to the experimental samples and served as the control adhesive for all the comparison tests in this research. All the adhesives tested in this study are categorized as mild self-etch adhesives according to Van Meerbeek (7) classification for the pH of adhesives; however, the pH of water-free adhesives on the tooth surface can not be exactly stated because it depends on the amount of available water for their ionization. In addition it has been recommended that water-free adhesives should be applied with a moist-bonding technique in order to acidify the adhesive to be capable of etching tooth substrates (16). For compensating the lack of pulp humidity and water in compositions of the experimental adhesives, blot-drying is used to create sufficient bond strength for water-free adhesives as considered in this study.

Nanolayering of phosphoric acid esters on dentin has been evaluated leading to the conclusion that nanolayering, which is a sign of chemical interaction with dental tissues, is affected by the concentration of MDP in the primer (5).

Yet, insufficient studies have been carried out to define the minimum effective concentration of the MDP functional monomer in 1-SEAs. It was shown in a study that minimizing water and functional monomer content of an adhesive could, to some extent, enhance shear bond strength (12).

In the present study, 0% and 5% MDP--containing adhesives had significantly lower bond strength than those with 10% and 15% concentrations of the monomer. Functional monomers can demineralize tooth structure when hydrolyzed, providing some micromechanical retention (20). However, increasing the MDP concentartion up to 15 % results in an increase µSBS. It seems that lower concentration of MDP monomer (0% and 5%) were not probably capable of proper demineralization of the dentin in the presence of unpredictable water available on dentin surface and lack of adequate chemical and/or micromechanical interactions between the adhesive and dentin structure. On the other hand, increasing the functional monomer concentration from 15% to 20% did not improve µSBS. MDP is a viscous monomer and it can be claimed that the increased concentration of functional monomer in the adhesive formulation results in a quite viscous resin blend with decreased penetration into the tooth substrate leading to lower bond strength (21,22). One could also argue that a higher concentration of MDP monomer results in a more hydrophilic adhesive mixture with more residual solvents, leading to a decrease in the physicomechanical characteristics of the hybrid layer (23). Although enamel bond strength was not assessed in the study, it was concluded that the enamel bond strength slightly increased with increasing the amount of MDP-calcium (MDP-Ca) salt, in contrast to the dentin (23,24).

Moreover, previous studies have revealed that the increased acidity of the adhesive leads to more adhesive fractures between the hydrophilic adhesive layer and the hydrophobic resin composite (25). Given the acidity of the studied adhesives, it seems that the maximum stress occurs more probably at the adhesive resin-composite resin interface than at the resin-dentin interface (11,13). The uneven stress distribution at the interface could affect the failure modes in the microshear bond strength test; however, in this study, fracture modes were mostly adhesive, exhibiting the validity of the performed test.

In scanning electronic microscopic, it was observed proper interfacial consistency and thicker adhesive layer in commercial adhesive than experimental adhesives that is may relate to its filler content (26). Therefore experimental adhesives are very prone to forming oxygen inhibition layer. So during the polymerization of the adhesive, most of the resin probably remains uncured. Then, when the composite is applied most of the adhesive is pushed away, thinning the adhesive even more. The resultant thin adhesive resin layer may then easily be copolymerized with the first layer of composite, and on a the flat surface this is no problem, but it would never use this in a cavity or in clinic, as it can not withstand polymerization shrinkage stress so well (27).

In the study, the highest DC% was observed in the CS3 group. This higher degree of conversion might be ascribed to the different chemical structure of the commercial adhesive in comparison to the experimental ones. The amount of Bis-GMA, which is a rigid monomer, and other monomers in formulations, filler contents, the viscosity of the adhesives, the amount and type of solvents and the photoinitiator system, which were different in the studied groups, may account for the differences observed in DC% of the adhesives (25,26). In addition, degree of conversion of an adhesive may be influenced by the concentration of acidic functional monomer (22). Adhesives with 0% and 5% functional monomer concentrations had lower degrees of conversion. Although we did not evaluate the viscosity of the experimental solutions here in tested, a previous study reported that there was a direct correlation between the viscosity of the adhesive and the degree of conversion (28).

‘Gel effect’ suggests that in higher viscosities the mobility of the polymer radicals is decreased concomitant with a reduction in chain termination, enhancing free radical propagation and vinyl conversion, which may lead to an increased polymerization rate (22,28).

DC% was observed to be lower in the E15 and E20 groups compared to the E5 group. The hydrophilic monomer present in an adhesive mixture can lower the vapor pressure of volatile solvents (29). It has been described that the higher functionality of a monomer, defined by the more double bonds in the molecule, accelerates the polymerization reaction, resulting in a faster onset of gelation and vitrification and forming a dense network of bonds. However the higher crosslink density reduces the conversion rate of the double bonds; thus many of them remain unreacted (30). The high concentration of functional monomer may affect the monomer conversion in the same way.

In the present study, adhesives with higher degrees of conversion demonstrated greater microshear bond strength values, except for the commercial adhesive (Clearfil S3 Bond) which did not have the highest microshear bond strength while showing the maximum degree of conversion.

This may be more related to the adhesive layer thickness, which was higher for Clearfil S3 Bond in comparison to experimental adhesives due to the filler content volume (26). We did not study the correlation between DC% and microshear bond strength because the conversion degrees of adhesives were mostly more than 60% which is considered as clinically acceptable polymerization (15,30). According to the results, the null hypothesis of the study was refuted.

We propose further studies on other variables using different functional monomers, solvents, initiators and fillers. Moreover, we evaluated only the dentin surface. Results might be quiet different in enamel and it might be supposed that adhesives with higher concentrations of functional monomer might act better in enamel substrate, which should be exactly studied.

## Conclusions

Under the limitations of this study, the results suggested that various MDP concentrations present in 1-SEAs affect the dentin shear bond strength and degree of conversion. According to the results of both tests and the ultra-structural morphology observed, 10% MDP seems to be the optimal functional monomer concentration for the examined water-free 1-SEAs.

## References

[1] Van Landuyt KL, Mine A, De Munck J, Jaecques S, Peumans M, Lambrechts P (2009). Are one-step adhesives easier to use and better performing?. Multifactorial assessment of contemporary one-step self-etching adhesives. J Adhes Dent.

[2] Van Landuyt KL, Snauwaert J, De Munck J, Peumans M, Yoshida Y, Poitevin A (2007). Systematic review of the chemical composition of contemporary dental adhesives. Biomaterials.

[3] Yoshida Y, Nagakane K, Fukuda R, Nakayama Y, Okazaki M, Shintani H (2004). Comparative study on adhesive performance of functional monomers. J Dent Res.

[4] Fukegawa D, Hayakawa S, Yoshida Y, Suzuki K, Osaka A, Van Meerbeek B (2006). Chemical interaction of phosphoric acid ester with hydroxyapatite. J Dent Res.

[5] Yoshihara K, Yoshida Y, Hayakawa S, Nagaoka N, Irie M, Ogawa T (2011). Nanolayering of phosphoric acid ester monomer on enamel and dentin. Acta Biomater.

[6] Van Landuyt KL, Yoshida Y, Hirata I, Snauwaert J, De Munck J, Okazaki M (2008). Influence of the chemical structure of functional monomers on their adhesive performance. J Dent Res.

[7] Van Meerbeek B, De Munck J, Yoshida Y, Inoue S, Vargas M, Vijay P (2003). Buonocore memorial lecture. Adhesion to enamel and dentin: current status and future challenges. Oper Dent.

[8] Li N, Nikaido T, Takagaki T, Sadr A, Makishi P, Chen J (2010). The role of functional monomers in bonding to enamel: acid-base resistant zone and bonding performance. J Dent.

[9] Van Landuyt KL, Snauwaert J, De Munck J, Coutinho E, Poitevin A, Yoshida Y (2007). Origin of interfacial droplets with one-step adhesives. J Dent Res.

[10] Tay FR, Pashley DH, Suh BI, Carvalho RM, Itthagarun A (2002). Single – step adhesive are permeable membranes. J Dent.

[11] Tay FR, Pashley DH, Yiu Ck, Sanares AM, Wei SH (2003). Factors contributing to the incompatibility between simplified - step adhesives and chemically - cured or dual - cured composites. Part I: single – step self – etching adhesive. J Adhes Dent.

[12] Kanehira M, Finger WJ, Ishihata H, Hoffmann M, Manabe A, Shimauchi H (2009). Rationale behind the design and comparative evaluation of an all-in-one self-etch model adhesive. J Dent.

[13] Tay FR, Pashley DH, Suh BI, Carvalho R, Miller M (2004). Single-step, self-etch adhesives behave as permeable membrans after polymerization.Part I.Bond strength and morphologic evidence. Am J Dent.

[14] Park J, Ye Q, Topp EM, Misra A, Kieweg SL, Spencer P (2010). Effect of photoinitiator system and water content on dynamic mechanical properties of a light-cured bisGMA/HEMA dental resin. J Biomed Mater Res A.

[15] Sakaguchi RL, Wiltbank BD, Shah NC (2004). Critical configuration analysis of four methods for measuring polymerization shrinkage strain of composites. Dent Mater.

[16] Van Landuyt KL, Mine A, De Munck J, Countinho E, Peumans M, Jaecques S (2008). Technique sensitivity of water-free one-step adhesives. Dent Mater.

[17] Tonetto MR, Pinto SC, Rastelli Ade N, Borges AH, Saad JR, Pedro FL (2013). Degree of conversion of polymer-matrix composite assessed by FTIR analysis. J Contemp Dent Pract.

[18] McDonough WG, Antonucci JM, He J, Shimada Y, Chiang MY, Schumacher GE (2002). A microshear test to measure bond strengths of dentin-polymer interfaces. Biomaterials.

[19] Scherrer SS, Cesar PF, Swain MV (2010). Direct comparison of the bond strength results of the different test methods: a critical literature review. Dent Mater.

[20] Moosavi H, Hariri I, Sadr A, Thitthaweerat S, Tagami J (2013). Effects of curing mode and moisture on nanoindentation mechanical properties and bonding of a self-adhesive resin cement to pulp chamber floor. Dent Mater.

[21] Ikemura K, Tay FR, Nishiyama N, Pashley DH, Endo T (2006). Design of new phosphonic acid monomers for dentaladhesives--synthesis of (meth) acryloxyalkyl 3-phosphonopropionates and evaluation of their adhesion-promoting functions. Dent Mater J.

[22] Oguri M, Yoshida Y, Yoshihara K, Miyauchi T, Nakamura Y, Shimoda S (2012). Effects of functional monomers and photo-initiators on the degree of conversion of a dental adhesive. Acta Biomater.

[23] Iwai H, Fujita K, Iwai H, Ikemi T, Goto H, Aida M (2013). Development of MDP-based one-step self-etch adhesive--effect of additional 4-META on bonding performance. Dent Mater J.

[24] Iwai H, Nishiyama N (2012). Effect of calcium salt of functional monomer on bonding performance. J Dent Res.

[25] Ikeda M, Tsubota K, Takamizawa T, Yoshida T, Miyazaki M, Platt JA (2008). Bonding durability of single-step adhesives to previously acid-etched dentin. Oper Dent.

[26] Van Dijken JW (2010). A prospective 8-year evaluation of a mild two-step self-etching adhesive and a heavily filled two-step etch-and-rinse system in non-carious cervical lesions. Dent Mater.

[27] Koga K, Tsujimoto A, Ishii R, Iino M, Kotaku M, Takamizawa T (2011). Influence of oxygen inhibition on the surface free-energy and dentin bond strength of self-etch adhesives. Eur J Oral Sci.

[28] Nunes TG, Garcia FC, Osorio R, Carvalho R, Toledano M (2006). Polymerization efficacy of simplified adhesive systems studied by NMR and MRI techniques. Dent Mater.

[29] Malacarne-Zanon J, Pashley DH, Agee KA, Foulger S, Alves MC, Breschi L (2009). Effects of ethanol addition on the water sorption/solubility and percent conversion of comonomers in model dental adhesives. Dent Mater.

[30] Cadenaro M, Antoniolli F, Codan B, Agee K, Tay FR, Dorigo Ede S (2010). Influence of different initiators on the degree of conversion of experimental adhesive blends in relation to their hydrophilicity and solvent content. Dent Mater.

